# Impact of the COVID-19 pandemic on patients with hepatocellular carcinoma in the West of Scotland: a cohort study

**DOI:** 10.1136/bmjgast-2023-001231

**Published:** 2023-11-20

**Authors:** Alistair Stewart McLaren, Johannes A Spoor, Douglas Cartwright, Gregory Naylor, Stephen Barclay, Matthew Priest, Srikanth Puttagunta, Kirsty Armstrong, Stuart Ballantyne, Adrian Stanley, Thomas R Jeffry Evans, West of Scotland HCC MDT, Kirsty Armstrong

**Affiliations:** 1 School of Cancer Sciences, College of Medical Veterinary and Life Sciences, University of Glasgow, Glasgow, UK; 2 CRUK Scotland Institute, Glasgow, UK; 3 Department of Gastroenterology, Glasgow Royal Infirmary, Glasgow, UK; 4 Gastrointestinal Cancers, Beatson West of Scotland Cancer Centre, Glasgow, UK; 5 Walton Liver Clinic, Glasgow Royal Infirmary, Glasgow, UK; 6 Department of Gastroenterology, Queen Elizabeth University Hospital, Glasgow, UK; 7 Department of Radiology, NHS Greater Glasgow and Clyde, Glasgow, UK; 8 School of Cancer Sciences, University of Glasgow, Glasgow, UK

**Keywords:** HEPATOCELLULAR CARCINOMA, COVID-19, CANCER EPIDEMIOLOGY

## Abstract

**Objective:**

The COVID-19 pandemic had an undoubted impact on the provision of elective and emergency cancer care, including the diagnosis and management of patients with hepatocellular carcinoma (HCC). Our aim was to determine the effects of the COVID-19 pandemic on patients with HCC in the West of Scotland.

**Design:**

This was a retrospective audit of a prospectively collated database of patients presented to the West of Scotland Multidisciplinary Team (MDT) between April and October 2020 (during the pandemic), comparing baseline demographics, characteristics of disease at presentation, diagnostic workup, treatment and outcomes with patients from April to October 2019 (pre pandemic).

**Results:**

There was a 36.5% reduction in new cases referred to the MDT during the pandemic. Patients presented at a significantly later Barcelona Cancer Liver Clinic stage (24% stage D during the pandemic, 9.5% pre pandemic, p<0.001) and with a significantly higher Child-Pugh Score (46% Child-Pugh B/C during the pandemic vs 27% pre pandemic, p<0.001). We observed a reduction in overall survival (OS) among all patients with a median OS during the pandemic of 6 months versus 17 months pre pandemic (p=0.048).

**Conclusion:**

The impact of the COVID-19 pandemic is likely to have contributed to a reduction in the presentation of new cases and survival among patients with HCC in the West of Scotland. The reason for this is likely multifactorial, but disruption of standard care is likely to have played a significant role. Resources should be provided to address the backlog and ensure there are robust investigation and management pathways going forward.

**WHAT IS ALREADY KNOWN ON THIS TOPIC**

- The COVID-19 pandemic led to an emphasis on acute care and an alteration in routine follow-up for patients with chronic illnesses.
- Detection at an earlier stage of hepatocellular carcinoma (HCC), prior to symptomatic presentation, will improve survival outcomes.
- Other regions of the UK and worldwide showed a reduction in presentation of the number of new cases of HCC.

**WHAT THIS STUDY ADDS**

- The COVID-19 pandemic has shown a reduction in overall survival among patients with HCC discussed in the West of Scotland despite the full range of treatment options being available, likely due to presentation at a later stage of illness.

**HOW THIS STUDY MIGHT AFFECT RESEARCH, PRACTICE OR POLICY**

- The backlog of cases not detected during the pandemic is likely to lead to patients presenting with more advanced HCC and a corresponding rise in morbidity and mortality.
- Increased resource should be made available to primary care, diagnostic services and secondary care to ameliorate the effects of the pandemic on patients with HCC.

## Introduction

The COVID-19 pandemic focused medical resources on acute and critical care, with a consequent impact on patients with chronic illnesses. Healthcare authorities aimed to reduce viral transmission by reducing face-to-face interactions in scheduled care. Patients also attended primary and secondary care less often, particularly for in-person consultations. These changes contributed to a decrease in cancer diagnoses.^1^ A recent national population-based modelling study showed an estimated increase in cancer deaths between 4.8% and 16.6%^2^ attributed to delays in diagnosis of treatable cancers. An additional increase in mortality is to be expected from suboptimal or delayed cancer treatment.^3^

A recent international survey of 76 institutions that evaluated hepatocellular carcinoma (HCC) and intrahepatic cholangiocarcinoma care found that 87% of centres had modified their clinical practice. A further 40% had modified their diagnostic procedures, 80% modified their screening programme and 50% cancelled curative or palliative treatments for liver cancer.^4^

In this retrospective analysis of a prospectively collated dataset, we examined the difference in outcomes for new patients referred to the West of Scotland HCC Multidisciplinary Team (MDT) and patients returning for discussion at the MDT to determine if there were significant differences during the COVID-19 pandemic compared with the previous year. We illustrate the impact of COVID-19 on HCC diagnosis, treatment and mortality.

## Methods

After Caldicott Guardian (a safeguard within National Health Service organisations to ensure proper use of confidential health information) approval, data was collected from the West of Scotland Hepatocellular Carcinoma MDT. This regional MDT discusses patients with suspected or confirmed HCC from 10 acute hospitals within 4 hospital boards serving a catchment of 2.5 million people (around 60% of Scotland’s population^5^). All patients with suspected HCC are expected to be referred for discussion of management as well as cancer registration purposes, and previous audit data suggests that greater than 80% of cases are discussed.^6^ Two cohorts were collected: those discussed during 1 April and 31 October 2019 (pre pandemic) and those discussed during 1 April and 31 October 2020 (pandemic). These dates were chosen for data collection for a comparable 6-month period, one prior to and one during the first wave of the COVID-19 pandemic.

Demographic and clinical data was collected at the time of discussion in MDT from the electronic patient record (EPR) accessed through Clinical Portal software (Orion Health, Auckland, New Zealand). A diagnosis of HCC was confirmed either radiologically by CT/MRI Liver Imaging Reporting and Data System (LI-RADS) criteria^7^ or histologically following biopsy. Patient outcomes were determined from individual patient EPRs.

Eastern Cooperative Oncology Group (ECOG) performance status^8^ was recorded by the referring clinician, with Child-Pugh Score^9^ calculated and alpha-fetoprotein (AFP) recorded from contemporaneous blood tests available at referral. The number of lesions and the maximum diameter of the largest lesion were calculated radiologically at the time of the MDT discussion. Barcelona Clinic Liver Cancer (BCLC) staging^10^ was retrospectively calculated from data collected at the time of the MDT discussion by the investigators. Information was recorded on whether a CT, MRI or surveillance ultrasound (US) had been performed prior to discussion at the MDT meeting to assess whether COVID-19 had an impact on availability of these services.

Statistical analysis was performed using R software (R foundation for statistical computing, Vienna, Austria). Statistical significance was assessed using Fisher’s exact test when comparing multiple variables and χ^2^ tests when comparing single variables. Log rank tests were used to assess statistical significance in comparison of survival outcomes using the Kaplan-Meier estimator, censoring data if patients were alive on 31 March 2022, 3 years after collection of pre pandemic data and 2 years after collection of pandemic data. Median follow-up of the pre pandemic group was 32 months and for the pandemic group was 21 months by the reverse Kaplan-Meier estimator.

### Patient and public involvement

No direct patient and public involvement was sought.

## Results

### Baseline characteristics

In total, 182 and 122 patients were discussed in the pre pandemic and pandemic periods, respectively. In total, 70 patients (35 pre pandemic, 35 pandemic) were excluded due to a lack of confirmation of HCC diagnosis, resulting in 236 patients (149 pre pandemic, 87 pandemic) for further analysis. Of these, 104 (69.8%) pre pandemic patients and 66 (75.9%) pandemic patients were new referrals.

Significant differences in baseline characteristic were seen between the two periods (table 1), with higher ECOG performance status, Child-Pugh scores and higher proportions of BCLC stage D in patients discussed during the pandemic. Hepatitis C was a more common aetiology of liver disease in the pre pandemic period. Other baseline characteristics were broadly similar, although there was a higher proportion of new male patients discussed during the pandemic than pre pandemic.

**Table 1:** Baseline characteristics of all and new patients presenting to MDT pre pandemic and during the pandemic

Characteristic	All			New		
	Pre pandemic, n=149‡	Pandemic, n=104‡	P value§	Pre pandemic, n=87‡	Pandemic, n=66‡	P value§
Age	69 (62–75)	69 (63–76)	0.60$	71 (62–76)	70 (63–77)	0.76¶
Child-Pugh			<0.001*			<0.001*
A	109 (73)	56 (54)		59 (68)	32 (48)	
B	39 (26)	37 (36)		28 (32)	24 (36)	
C	1 (0.7)	11 (11)		0 (0)	10 (15)	
ECOG PS at referral			0.024*			0.23*
0	67 (48)	30 (29)		37 (43)	18 (27)	
1	39 (28)	34 (33)		23 (26)	19 (29)	
2	20 (14)	21 (21)		15 (17)	15 (23)	
3	13 (9.4)	16 (16)		11 (13)	13 (20)	
4	0 (0)	1 (1.0)		0 (0)	1 (1.5)	
NA				1 (1.1)	0 (0)	
Sex			0.14†			**0.035†**
F	29 (19)	13 (12)		19 (22)	6 (9.1)	
M	120 (81)	91 (88)		68 (78)	60 (91)	
Aetiology of HCC			0.002*			0.002*
Hepatitis B	1 (0.7)	7 (6.7)		1 (1.1)	6 (9.1)	
Hepatitis C	33 (22)	11 (11)		19 (22)	4 (6.1)	
Non-cirrhotic	1 (0.7)	0 (0)		0 (0)	0 (0)	
Non-viral	114 (77)	86 (83)		67 (77)	56 (85)	
BCLC stage			<0.001*			0.019*
A	29 (20)	14 (13)		14 (16)	7 (11)	
B	21 (14)	7 (6.7)		9 (10)	4 (6.1)	
C	76 (51)	58 (56)		50 (57)	33 (50)	
D	14 (9.5)	25 (24)		11 (13)	22 (33)	
O	8 (5.4)	0 (0)		3 (3.4)	0 (0)	

p values in bold are significant.

*Person’s χ^2^ test.

†Fisher’s exact test.

‡n (%); median (IQR).

§Fisher’s exact test; Pearson’s χ^2^ test; and Wilcoxon rank sum test.

¶Wilcoxon rank sum test.

BCLC, Barcelona Clinic Liver Cancer staging; ECOG PS, Eastern Cooperative Oncology Group Performance Status; HCC, hepatocellular carcinoma; MDT, Multidisciplinary Team.

### Features of disease at presentation

AFP, size of maximum tumour diameter and the number of lesions within the liver were recorded at the time of the MDT discussion. No significant differences were identified, though there was a trend towards a higher median AFP (8.0 ng/mL, IQR 0–64 ng/mL pre pandemic vs 16.0 ng/mL, IQR 4–232 ng/mL during the pandemic, p*=*0.12 Wilcoxon rank sum test), and a longer median maximum diameter of the largest lesion (30 mm, IQR 18–63 mm in 2019 vs 41 mm, IQR 22–75 mm in 2020, p*=*0.14), though there was no difference observed in the number of lesions at presentation, as per table 2. These trends were replicated among new patients, but not return patients, and did not reach statistical significance in either group. The two patients identified with no lesions were return patients being discussed in MDT following treatment of a previous HCC.

**Table 2:** Patients with a defined number of HCC lesions within the liver at the time of presentation to MDT pre pandemic and during the pandemic

Characteristic	All			New		
	Pre pandemic, n=149*	Pandemic, n=104*	P value†	Pre pandemic, n=87*	Pandemic, n=66*	P value†
Number of lesions			0.43			0.87
0	2 (1.3)	0 (0)		0 (0)	0 (0)	
1	72 (48)	49 (48)		47 (54)	32 (48)	
2–3	48 (32)	29 (28)		20 (23)	14 (21)	
>3	30 (20)	25 (24)		20 (23)	20 (30)	

*n (%).

†Fisher’s exact test.

### Diagnostic workup and management

Among all patients, similar proportions had diagnostic investigations pre pandemic as during the pandemic. However, there was a statistically significant difference in new patients having triple-phase CT scan of the liver as part of their diagnostic pathway, with 57% of patients having this investigation in 2020 compared with 74% in 2019 (p=0.018). Pre pandemic, 60 patients (40.3%) had both triple-phase CT scan and MRI of the liver in their workup prior to MDT discussion (40 new, 46%, and 20 return, 32.3%), and during the pandemic, 34 patients (32.7%) had both in their workup (22 new, 33.3%, and 12 return, 31.6%). A comparison of investigations performed can be seen in table 3. There were also trends in difference in how new patients were managed in 2020 compared with 2019 but these were not statistically significant with a higher proportion (55% vs 39%) managed with best supportive care (BSC) and a lower proportion (5.8% vs 19%) managed with microwave ablation. Table 4 has proportions of management outcomes selected from the MDT.

**Table 3:** Number and proportions of diagnostic investigations performed for patients presented in the MDT pre pandemic and during the pandemic

Characteristic	All			New		
	Pre pandemic, n=149	Pandemic, n=104	P value†	Pre pandemic, n=87	Pandemic, n=66	P value†
US surveillance	18 (12)	10 (9.6)	0.54	13 (15)	10 (15)	0.97
Triple-phase CT scan	104 (70)	61 (59)	**0.067**	63 (72)	36 (55)	**0.022**
MRI liver	93 (62)	55 (53)	0.13	57 (66)	36 (55)	0.17
Biopsy	22 (15)	12 (12)	0.46	18 (21)	11 (17)	0.53

p values in bold are significant

*n (%).

†Pearson’s χ^2^ test.

MDT, Multidisciplinary Team; US, ultrasound.

**Table 4:** Management decisions made for patients presenting to the MDT in 2019 and 2020

Characteristic	All			New		
	Pre pandemic, n=149	Pandemic, n=104	P value†	Pre pandemic, n=87	Pandemic, n=66	P value†
Outcome			0.11			**0.069**
BSC	47 (32)	51 (49)		36 (41)	38 (58)	
Irreversible electroporation	1 (0.7)	0 (0)		0 (0)	0 (0)	
Microwave ablation	24 (16)	10 (9.7)		18 (21)	4 (6.1)	
Resection	2 (1.3)	2 (1.9)		2 (2.3)	2 (3.0)	
SACT	23 (15)	10 (9.6)		12 (14)	6 (9.1)	
SIRT	1 (0.7)	0 (0)		1 (1.1)	0 (0)	
Surveillance	12 (8.1)	5 (4.8)		2 (2.3)	0 (0)	
TACE	36 (24)	24 (23)		14 (16)	15 (23)	
Transplant	3 (2.0)	2 (1.9)		2 (2.3)	1 (1.5)	

p values in bold are significant.

*n (%).

†Fisher’s exact test.

BSC, best supportive care; MDT, Multidisciplinary Team; SACT, systemic anticancer therapy; SIRT, selective internal radiation therapy; TACE, transarterial chemoembolisation.

### Survival

**Figure 1:**
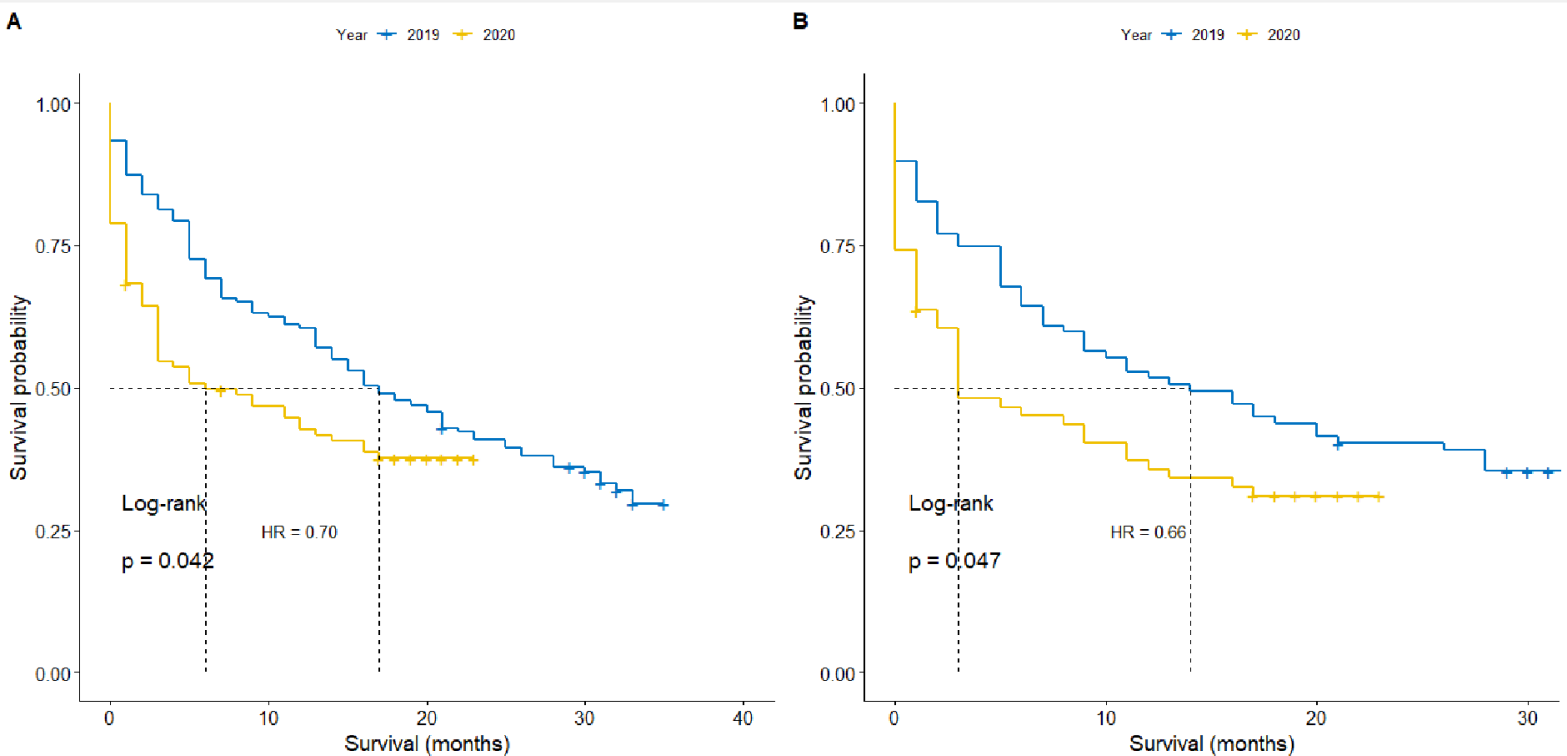
Overall survival from all-cause mortality for (A) all and (B) new patients presenting to the West of Scotland Hepatocellular Carcinoma Multidisciplinary Team prior to (blue line) and during the pandemic (yellow line).

Median overall survival (OS) from all-cause mortality timed from date of discussion at MDT was 17 months (95% CI 13 to 23 months) in the pre pandemic group compared with 6 months (95% CI 3 to 16 months) in 2020 (p=0.042) (figure 1A). The HR for death was 0.70 (95% CI 0.51 to 0.98, p=0.035) in the pre pandemic group compared with during the pandemic, and 12-month survival was 60.4% pre pandemic compared with 42.6% in the pandemic. New patients had a median OS of 14 months (95% CI 9 to 28 months) pre pandemic compared with 3 months (95% CI 2 to 12 months, p=0.048) in 2020 (figure 1B). The HR for death was 0.66 (95% CI 0.44 to 0.98, p=0.04) pre pandemic compared with during the pandemic for new patients. 12-month survival of new patients presenting to the MDT was 52% prior to the pandemic and 36% during the pandemic. In return patients that had been previously discussed, median OS was 21 months (95% CI 15 to 30 months) pre pandemic and 16 months during the pandemic (95% CI 4 months to Not Reached (NR), p=0.64). HR for death was 0.87 (95% CI 0.5 to 1.51, p=0.63) during the pandemic compared with prior to the pandemic. Twelve-month survival in return patients was 73% pre pandemic and 55% during the pandemic.

## Discussion

This study explores the impact of the COVID-19 pandemic on outcomes of patients with HCC in the West of Scotland. There was a 36.5% reduction in new cases of HCC discussed at the MDT, which contradicts the evidence for rising incidence of HCC in the UK.^11^ This reduction in incidence during COVID-19 has been seen in centres in Asia^12^ and in the North East of England.^13^ This is in keeping with the fall in diagnoses of all cancers seen in the UK during the first wave of the COVID-19 pandemic.^1^ In our data, there was a significant reduction in patients diagnosed with hepatitis C virus-driven HCC (HCV-HCC) during the pandemic compared with the year prior, likely due to the reductions in HCV-related HCC subsequent to the scale up of direct acting antivirals.^14^

Of concern, there was decreased survival in patients presenting with HCC during the COVID-19 pandemic compared with a similar period in the previous pandemic-free year. This held true for all patients but was most marked in patients presenting to the MDT for the first time with new diagnoses of HCC. There may be a number of explanations for this, and we acknowledge there are several hypotheses that may fit. One is that patients are presenting later during the pandemic than before. This would explain our finding of significantly higher BCLC stage and Child-Pugh Score at presentation and a trend towards a larger median diameter of the largest tumour lesion and AFP during the pandemic. Another explanation is that patients may have died from COVID-19 during the pandemic, as patients with chronic liver disease (CLD) and cancer were more vulnerable.^15 16^ We were unable to collect reliable diagnostic and survival data on COVID-19, but a contemporaneous study in the North of England^13^ suggests that rates of COVID-19 in this population were low, which may have been positively impacted by the ‘shielding’ policy adopted by the UK government. A higher but not significant proportion of patients received BSC in 2020 compared with 2019, which may reflect presentation at a later stage of both liver disease and cancer stage, as access to other therapies for radical and palliative management of HCC were not affected.

Significantly fewer new patients during the COVID-19 pandemic had triple-phase CT scans of the liver performed compared with the year prior. This was not seen in patients returning for discussion in the MDT, where rates were similar during the pandemic. Access to elective radiology services was diminished at the start of the COVID-19 pandemic,^17 18^ which coupled with the cancellation of elective services. This may have led to pragmatic decision-making to forego triple-phase CT scan where MRI (local standard first line investigation of focal liver lesions) was diagnostic of HCC. Another possible explanation is that additional imaging investigations were not performed in patients with worse BCLC stage and Child-Pugh scores as these investigations would not change management in these patients in whom only BSC was feasible. Although there has been a reported drop in HCC surveillance worldwide,^19^ access to screening US does not seem to have been affected, with similar numbers of new cases detected on US surveillance prior to and during the pandemic. Real-life adherence rate to US surveillance in a population at risk for HCC has been reported to be around 39%,^20^ lower than an adherence rate of 76.6% in the West of Scotland,^21^ so this is unlikely to have affected the number of cases first detected on US surveillance in this population.

Limitations of this study include the relatively low number of patients in the study, which likely masks significant difference seen between the 2019 and 2020 populations, especially in terms of survival. As a single-centre study, although from a large patient population across several hospitals, it is hard to infer how services may have been affected in other institutions elsewhere in Scotland, the UK and worldwide, with differing responses to the COVID-19 pandemic at a local as well as a national level. Having limited data on cause of death in our patient population has also not allowed us to examine if the lower survival in the 2020 population was as a direct result of COVID-19 infection or its sequelae.

Nevertheless, results from this study raise concerns of the adverse effects of the pandemic on patients with HCC. There are likely to still be cases of undiagnosed HCC in the community despite efforts to restart elective care, due to the backlogs that primary and secondary care are experiencing because of the COVID-19 pandemic. The decreased survival of patients observed during the COVID-19 pandemic may continue to be the case for years to come, and efforts should be made to ameliorate this, with increased resource in secondary care and imaging to allow for earlier detection. The later diagnosis of HCC, if it were to continue, will also have an impact on interventional radiology, oncology and palliative care services that provide palliative intervention for HCC and will also require resourcing.

Vulnerable groups of patients with chronic disease had adverse outcomes with the emphasis on acute care during the COVID-19 pandemic, as the evidence from this study shows in patients with HCC. It is of vital importance that they do not remain further neglected as healthcare recovers from the global impact of the pandemic.

## Data Availability

Anonymised data will be available upon reasonable request to the corresponding author.
